# A152 THE RISK OF UNFAVORABLE HISTOLOGIC FEATURES, LYMPH NODE METASTASIS AND METACHRONOUS ADVANCED NEOPLASIA IN PATIENTS WITH COLORECTAL INTRAMUCOSAL CARCINOMA

**DOI:** 10.1093/jcag/gwad061.152

**Published:** 2024-02-14

**Authors:** E Medawar, I Popescu Crainic, R Djinbachian, M Zarandi-Nowroozi, M Taghiakbari, P Marques, D Kaufman, D von Renteln

**Affiliations:** University of Ottawa Faculty of Medicine, Ottawa, ON, Canada; Universite de Montreal Faculte de Medecine, Montreal, QC, Canada; Universite de Montreal Faculte de Medecine, Montreal, QC, Canada; Universite de Montreal Faculte de Medecine, Montreal, QC, Canada; Universite de Montreal Faculte de Medecine, Montreal, QC, Canada; McMaster University, Hamilton, ON, Canada; Universite de Montreal Faculte de Medecine, Montreal, QC, Canada; Universite de Montreal Faculte de Medecine, Montreal, QC, Canada

## Abstract

**Background:**

Very few studies have described the risk of unfavorable histologic features (UHFs), lymph node involvement (LNI), distant metastasis (DM) and metachronous advanced neoplasia (MAN) after the detection of colorectal intramucosal carcinoma (IMC).

**Aims:**

This study aimed to determine the risk of UHFs, LNI, DM and MAN in patients with IMC, compared to high-grade dysplasia (HGD) or T1 colorectal cancer (CRC).

**Methods:**

A retrospective cohort study was conducted. Through a pathology database search, we identified all consecutive patients with HGD (epithelial) (n=908) or either IMC (lamina propria or muscularis mucosa) or T1 CRC (submucosa) (n=496) diagnosed between 2010 and 2019 at the University of Montreal Hospital Center. We excluded patients with age ampersand:003C40 or ampersand:003E90, IBD, hereditary CRC or post-neoadjuvant therapy. Primary outcome was the risk of UHF, LNI and DM in IMC vs T1. Secondary outcome was the risk of MAN (metachronous advanced adenoma or advanced serrated lesion or CRC) in the IMC group, compared to HGD and T1. Files were reviewed for demographics and index and follow-up findings. The metachronous analysis excluded follow-ups ampersand:003C6 months, no follow-up or previous CRC. Follow-up continued until MAN or last colonoscopy within 10 y. Variables were compared between groups using ANOVA, chi-squared tests or Kruskal-Wallis tests, followed by post-hoc tests. We performed multivariate Cox regressions, adjusting for age, sex, family history of CRC, main lesion location, villous histology, adenomas ≥10 mm, and number of adenomas.

**Results:**

The primary cohort included 90 patients with IMC (mean age 69.4 y., 44.4% female) and 207 with T1 CRC (mean age 69.8 y., 43.5% female), as well as 797 with HGD. The rates of lymphatic invasion, vascular invasion, intermediate differentiation/grade and mucinous histology were 2/90, 1/90, 7/90 and 4/90 in the IMC group compared to respectively 30/207 (2.2% vs 14.5%, pampersand:003C0.01), 15/207 (1.1% vs 7.2%, p=0.06), 68/207 (7.7% vs 32.9%, pampersand:003C0.01) and 15/207 in the T1 group (4.4% vs 7.2%, p=0.52). No other UHFs were present. No patients with IMC had LNI or DM.

In the metachronous outcomes analysis, 464 patients were eligible (mean age 67.1 y., 42.2% female, median follow-up 3.0 y.): 274 with HGD, 58 with IMC and 132 with T1 CRC. Compared to HGD, patients with IMC had a lower likelihood of MAN (HR 0.46, 0.22-0.96), and T1 CRC had a similar likelihood of MAN (HR 0.65, 0.41-1.02). Compared to IMC, T1 CRC had a similar likelihood of MAN (HR 1.39, 0.63-3.09).

**Conclusions:**

Patients with colorectal IMC have a small risk of UHFs, but LNI and DM were not identified in this cohort study and have not been reported. Patients with IMC and T1 CRC do not have an increased risk of MAN compared to patients with HGD.

**Funding Agencies:**

None

